# Determining the maximum length of logical rules in a classifier and visual comparison of results

**DOI:** 10.1016/j.mex.2020.100846

**Published:** 2020-03-03

**Authors:** José A. Castellanos-Garzón, Ernesto Costa, José Luis S. Jaimes, Juan M. Corchado

**Affiliations:** a IBSAL/BISITE Research Group, University of Salamanca, Edificio I+D+i, 37007 Salamanca, Spain; b Department of Computer Engineering, Center of Informatics and Systems, Faculty of Science and Technology, University of Coimbra, Pólo II - Pinhal de Marrocos, 3030-290 Coimbra, Portugal; c Instituto Universitario de Estudios de la Ciencia y la Tecnología (ECYT), University of Salamanca, Edificio I+D+i, 37007 Salamanca, Spain

**Keywords:** Machine learning, Logical rule induction, Data mining, Supervised learning, Evolutionary computation

## Abstract

Supervised learning problems can be faced by using a wide variety of approaches supported in machine learning. In recent years there has been an increasing interest in using the evolutionary computation paradigm as the classifier search method, helping the technique of applied machine learning. In this context, the knowledge representation in form of logical rules has been one of the most accepted machine learning approaches, because of its level of expressiveness. This paper proposes an evolutionary framework for rule-based classifier induction and is based on the idea of sequential covering. We introduce genetic programming as the search method for classification-rules. From this approach, we have given results on subjects as maximum rule length, number of rules needed in a classifier and the rule intersection problem. The experiments developed on benchmark clinical data resulted in a methodology to follow in the learning method evaluation. Moreover, the results achieved compared to other methods have shown that our proposal can be very useful in data analysis and classification coming from the medical domain.

• The method is based on genetic programming techniques to find rules holding each class in a dataset.
• The method is approached to solve the problem of rule intersection from different classes.
• The method states the maximum length of a rule to generalize.

## Graphical abstract

**Figure:**
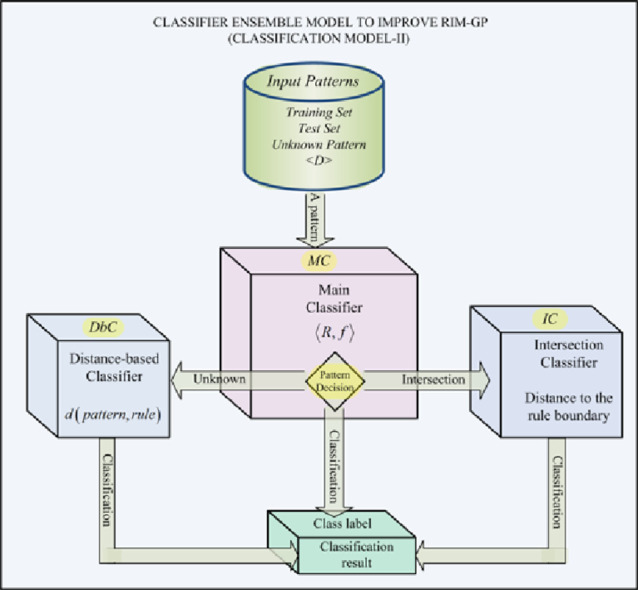

**Table:** Specification Table

Subject Area	*Computer Science*
More specific subject area:	*Computer science applied to medicine*
Method name:	*RIM-GP: Rule Induction Method based on Genetic Programming*
Name and reference of original method:	*Castellanos-Garzón, J. A., Costa, E., Jaimes, J. L. S., Corchado, J. M., An evolutionary framework for machine learning applied to medical data, Knowledge-Based Systems, Elsevier, 2019*
Resource availability:	*Center for Machine Learning and Intelligent Systems,* *http://archive.ics.uci.edu/ml/datasets/*

## Method details

The exponential growth of the amount of available medical data raises the problems of efficient storage and management of information as well as disclosing useful information from the data. The problem above is a challenge in computational medicine, claiming the development of methods and tools able to transform data into medical knowledge on the underlying mechanism. Those tools (methods) allow us to go beyond a simple description of the data and provide knowledge in form of models. Through this data abstraction involving a model, we will be able to obtain predictions of systems [1], [2], [3].

There are several medical domains where machine learning techniques have been applied to discover knowledge, such as, diagnosis and prognosis. The goal of machine learning in the context of diagnosis and prognosis is knowledge discovery needed to interpret the gathered information. The knowledge representation in the form of rules provides the expert with an explanation of the decision [4,5]. In this sense, evolutionary techniques (as genetic programming) are well suited to solve these kinds of problems due to their ability to carry out searches in extremely complex spaces. Machine learning constitutes the scene on which genetic programming can look for new relationships and validation of proposed hypotheses [6,7].

**Fig. 1:**
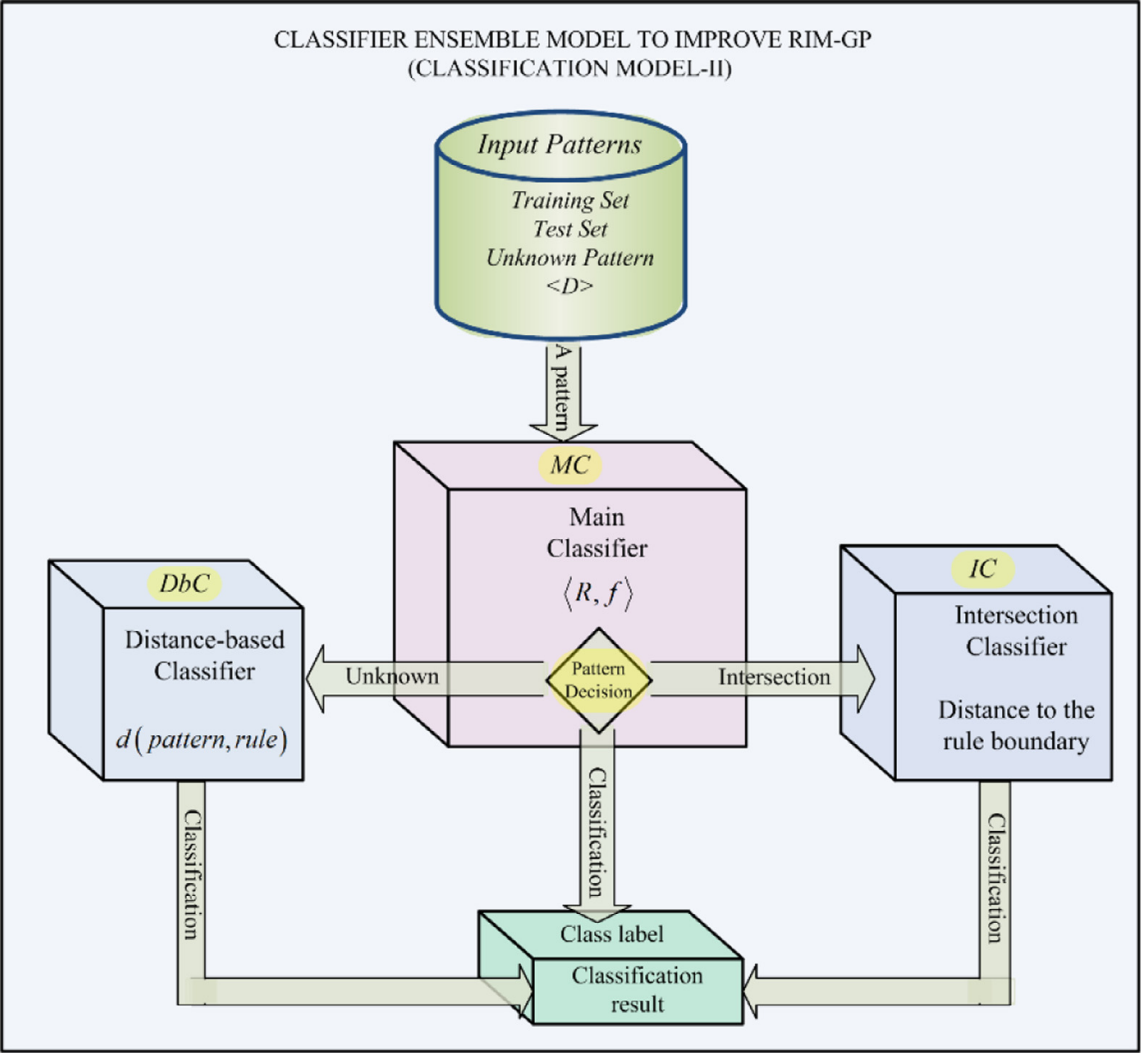
An ensemble-based classification model (RIM-GP*) consisting of three classifiers covering each region in the search space. MC is the rule based main classifier which generates rule intersection. IC is the intersection classifier, which is based on rule boundary. Finally, DbC is a distance-based classifier, which covers unknown regions (of the pattern space) for MC and IC.

According to all the previously explained, we present an evolutionary framework inducing rule-based classifiers [9]. This proposal uses genetic programming as an evolutionary technique to induce classification rules of type IF/THEN. Even though our proposal follows the sequential covering technique, a well-known one to render rules, it is still a novel approach. That is, we have introduced new fitness functions and genetic operators, a local search strategy to refine the found solutions. As a result of the contributions above, a new ensemble classifier method has been proposed to deal with the rule intersection problem, Fig. 1. Moreover, we have outlined several theoretical results with great practical implications related to the learning algorithm and its solution-classifiers.

The ensemble-based classification model shown in Fig. 1 faces the rule intersection problem by providing three classifiers. Each of these classifiers deals with complementary regions in the search space, i.e., the MC classifier almost covers the whole search space whereas the IC and DbC classifiers cover intersection and unknown regions respectively. Then, the proposed framework has been tested and validated on four clinical datasets, which are well-known in the literature. The achieved results have been very promising and competitive when compared with those from other methods. Considering all the above, we can say that our approach has proven to be very effective when used on clinical data. Finally, we can summarize the main contributions (highlights) as follows:

• An evolutionary framework for rule-based classifier induction.
• Genetic programming for logical rule induction.
• Finding the maximum size of a rule based on the data dimension.
• An ensemble classifier oriented to solve the rule intersection problem.
• Rule-based classification applied to clinical data.

### Rule generalization and size: antecedents of Theorem 1, maximum rule length (Appendix-B)

**Fig. 2:**
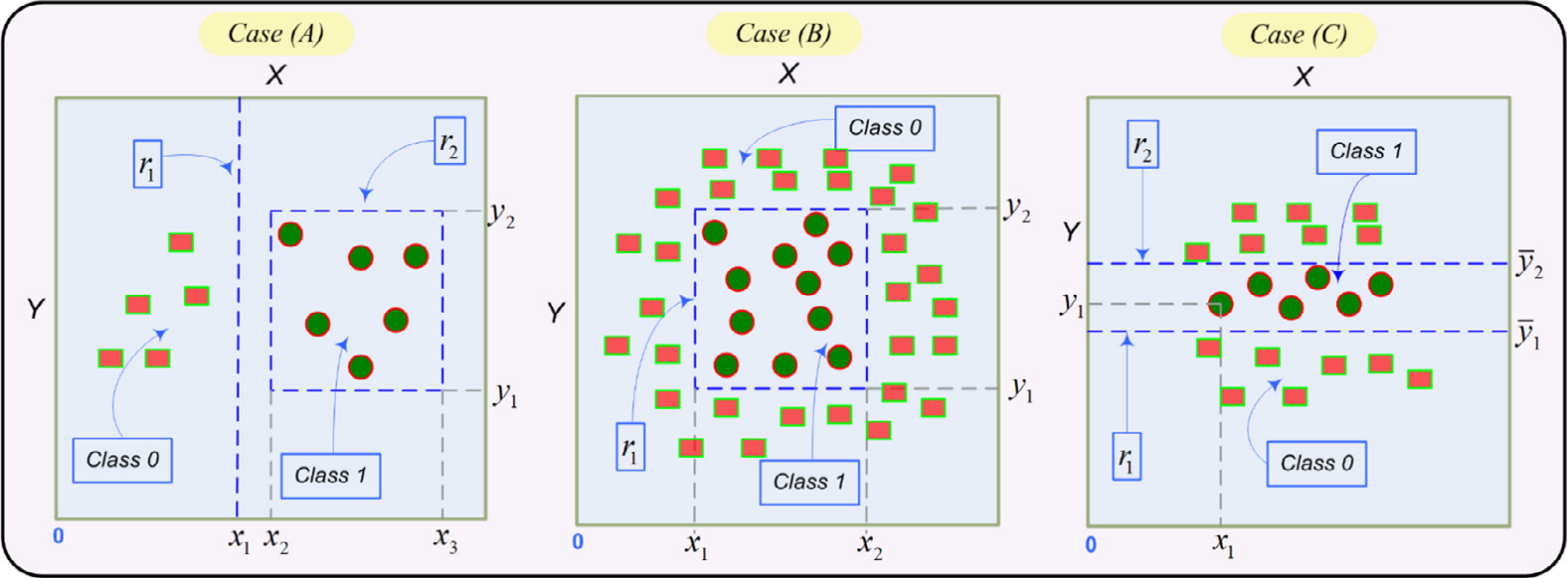
Three hypothetical cases of rule-based binary classification showing the concepts of rule generalization and overfitting onto plane X-Y. r1 and r2 represent rules. x1, x2, x3 and y1, y2, y1, y2 mean coordinates (or attribute values of a data set) for attributes *x* and *y* respectively onto the plane.

This section deals with the rule generalization problem starting from a two-dimensional search space such as, the examples given in Fig. 2. The intention of such examples is to determine when a rule generalizes and build an approximated interval for the maximum length of a logic rule. Fig. 2 will be the guide to reach the purpose above. To show concepts in an easy way, we represent rules only through their antecedents. The class of a rule will be explicitly given when needed, so this should not create confusion with respect to the rule definition given in Section 3 of the paper.

Then, from a visual standpoint, rule r1: (*x* ≥ x1) in Fig. 2-Case-(A) shows the generalization concept (assuming that we are classifying Class-1) whereas rule r2: (*x* ≥ x2) ∧ (*x* ≤ x3) ∧ (*y* ≥ y1) ∧ (*y* ≤ y2) does not generalize enough in Class-1 according to the point distribution given in Class-0. Now notice that rules r1 and r2 in Case-(A) represent two major cases, i.e., r1 represents the most general case that a rule can reach onto the plane, a single literal (a straight line). In contrast, r2 represents the least general case from all cases generalizing onto the plane, that is, four literals represent the maximum number of literals to generalize (a rectangle). Therefore, making an analogy with the above, the most general case separating two classes in a multidimensional space is a hyperplane region and conversely, a hyperrectangle region is the least general case (2×d literals, where d is the dimension). Hence, rules able to generalize represent subshapes of hyperrectangles.

Now for Case-(B) in Fig. 2, note that a rule as r1 which generalizes in Case-(A), no longer applies to Case-(B) because it overfits. Conversely, a rule as r2 overfitting in Case-(A), now generalizes in Case-(B). Thus, according to the data distribution given in Case-(B), rule r1 of this case, generalizes. So, generalization or overfitting also depends on the data distribution and how classes are interrelated in the training set. Namely, despite the fact that a straight line is the most general case to separate two classes onto the plane, it does not generalize in Case-(B) whereas a rectangle being the least general case, generalizes in Case-(B). Note also that rule set {r′1: (*x* ≤ x1) ∧ (*y* ≤ y1), r′2: (*x* ≥ x2) ∧ (*y* ≥ y2)} defined for Class-0 in Case-(B) generalizes, although two rules have been needed.

Case-(C) in Fig. 2 displays a more complex case where a rule is not enough to cover a class if we want to generalize. Class-0 can be classified by two rules as given in set R:= {r1: (*y* ≤ y1), r2: (*y* ≥ y2)}. But it can also be classified by a single rule discriminating patterns from Class-1. Namely, assume that the six patterns displayed in Class-1 have attribute values (xi, yi) where i ∈ [1,6], then we can build rule r3: (x ≠ x1)∧(y ≠ y1)∧· · ·∧(x ≠ x6)∧(y ≠ y6) also classifying Class-0. Although at first glance, both options (set R and rule r3) generalize in Class-0, R generalizes much more than r3 since R does not classify patterns whose attribute values y ∈ (y1, y2). Such patterns with attributes in (y1, y2) should be classified in Class-1 according to the point distribution of Case-(C). Contrasting set R, r3 does classify patterns considered as Class-1 (and different from those visualized by this class in Case-(C)) in Class-0. Hence, we can say rule r3 overfits. Thus, a greater number of rules in a classifier such as set R, does not necessarily imply that it is less efficient. Additionally, rule r3 also tells us that if a classifier has been forced to learn a single rule per class while classes are interrelated (as often happens: Case-(B) and (C) in Fig. 2), likely, the found rules will be as r3.

After studying the graphical representation of classification rules, we can now proceed to study rules from the analytical point of view, i.e., from their structures as a conjunction of literals pointing to a class. The first analysis to be performed is on the number of literals of a rule. A rule seen as a conjunction of literals where each literal is constraint imposed on the pattern space to classify the target class, it holds that the smaller the number of literals the more general the rule. Note that an increase of the number of literals (or the number of constraints), diminishes the rule coverage and so it tends to overfit. Hence, for a classifier generalizes, the number of literals for each of its rules must be as small as possible.

Now, it only remains to analyze the comparison operators used in the literals of a rule in search of an interval for the maximum rule length. As shown in rule visual analysis in Fig. 2, comparison operators {<, ≤, >, ≥} tend to generalize whereas operators {≠} tend to overfit (see analysis on operator ≠ in Fig. 2-Case-(C)). In fact, the case with the highest overfitting is the trivial one, i.e., a set of rules learned from a class, where each rule represents one and only one pattern of the class. Then, literals in each rule are formed by equalities (with operator =) between attributes and their values given by the pattern represented in the rule. In this case, every rule has d literals such as, the dimension of the pattern space. In addition, the number of rules matches the size of the class. Then, based on the above, it follows that the number of literals using operators {≠} in a rule should be as small as possible.

A consequence from the rule analysis carried out in this section is that we can fix the maximum length of a rule in the range [2d, kd]. 2d has been achieved from the analysis of Case-(A) and kd from the one of Case-(C) in Fig. 2. k is the number of patterns in the class opposite to the one that is being learned (see the previous analysis given for rule r3 in Case-(C)). The formal proof of this result is given in Appendix-B by Theorem 1 of the paper.

### Visual interpretation of the results given in Section 4, Table B.2

This section performs visual representations on the data sets (Dataset#1, #2, #3 and #4 from Section IV in the paper) used in this research, in an effort to explain the results given in Section IV-A of the paper. So, the aim of this section is to provide both a data distribution in the space and a two-dimensional map of the data and their classes. This will allow us to obtain an interpretation of the results reached by our proposal, RIM-GP.

In that sense, we provide two visualizations for each data set, that is, a 3D-scatterplot visualization where the data dimensionality as been reduced to three dimensions by applying principal component analysis (PCA). This allows us to display a whole data set and each class of it, separately. The second visualization is a heatmap of the data sets, i.e., a color map where colors are proportional to the values given by the data set. This way, each class in the data set can be differentiated by its color tonality. The 3D-VisualCluster tool given in [8] has been used to achieve both visualizations and moreover, it standardizes the data to mean 0 and variance 1.

**Fig. 3:**
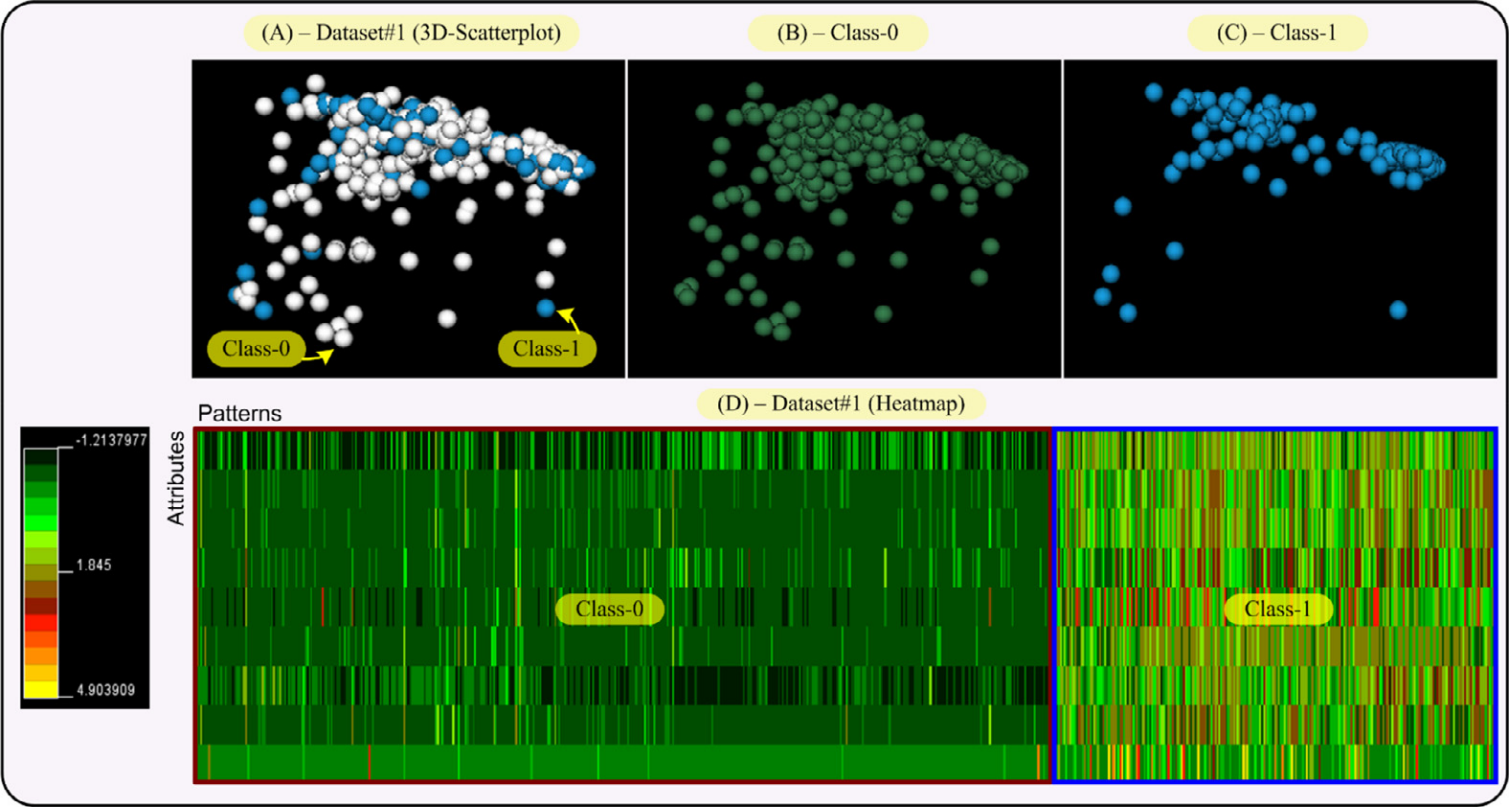
3D-scatterplot and heatmap visualizations for Dataset#1 (Breast Cancer Data). Each class in the scatterplot has a different color and the color scale for the heatmap goes from dark green (value: −1.20), passing by red (value: 1.84), to yellow (value: 4.89). Each class in Dataset#1 has also been framed on the heatmap. Attributes and patterns of the data set have been represented on the heatmap as rows and columns respectively. (For interpretation of the references to color in this figure legend, the reader is referred to the web version of this article.)

To show the visual results, we begin by showing Fig. 3 which displays views of Dataset#1. By analyzing this figure with respect to the Dataset#1 results in Table B.2 of the paper, the first thing we can say is that according to the 3D-scatterplot, most points from Dataset#1 form a compact region. Thus, the classes of Dataset#1 also form compact regions, meaning that the pattern-points from each class are very close together. This fact can facilitate the RIM-GP task of separating both classes through rules if the classes are not very interrelated. Reinforcing the criterium above, note that the color distribution of the heatmap of Dataset#1, shows that Class-0 and Class-1 are perfectly separable. Namely, pattern values shown by Class-0 in the heatmap are under 0.32 (dark green colors) whereas most values in Class-1 are above 0.32 (light green, red and yellow colors). Therefore, the structure of Dataset#1 displayed by Fig. 3 has allowed RIM-GP to find classifiers with the least number of rules and the smallest intersection (see Dataset#1 results in Table B.2 of the paper) compared with the other data sets. Hence, the RIM-GP classifiers have achieved the best accuracy values on Dataset#1, i.e., a mean accuracy of 89.94%.

**Fig. 4:**
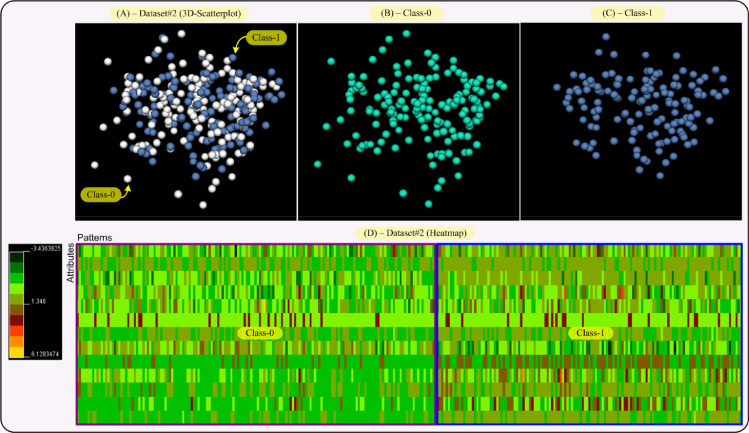
3D-scatterplot and heatmap visualizations for Dataset#2 (Cleveland Heart Disease Data). Each class in the scatterplot has a different color and the color scale for the heatmap goes from dark green (value: −3.43), passing by red (value: 1.35), to yellow (value: 6.13). Each class in Dataset#2 has also been framed on the heatmap. (For interpretation of the references to color in this figure legend, the reader is referred to the web version of this article.)

Fig. 4 shows both, 3D-scatterplot and heatmap for Dataset#2. As displayed on the scatterplot (Fig. 4-(A, B and C)), Dataset#2 is less compact than Dataset#1. Namely, data are more scattered in the space. In addition, pattern-points from both classes are more interrelated, Fig. 4 (A and D). This is best shown in the heatmap visualization, Fig. 4-(D). Note that both classes in the heatmap share the same colors, even though Class-1 has a higher tendency to brown color tonalities than Class-0, which tends to green color tonalities. This means that although the classes share similar colors, there is certain separability between them, but it is much less than the one for Dataset#1. Thus, the RIMGP task of separating both classes makes more difficult. Consequently, there will be an increment of the number of rules in classifiers and number of patterns in the intersection. The latter resulted in a reduction of the accuracy of RIM-GP classifiers using Dataset#2, as shown in Table B.2 of the paper.

**Fig. 5:**
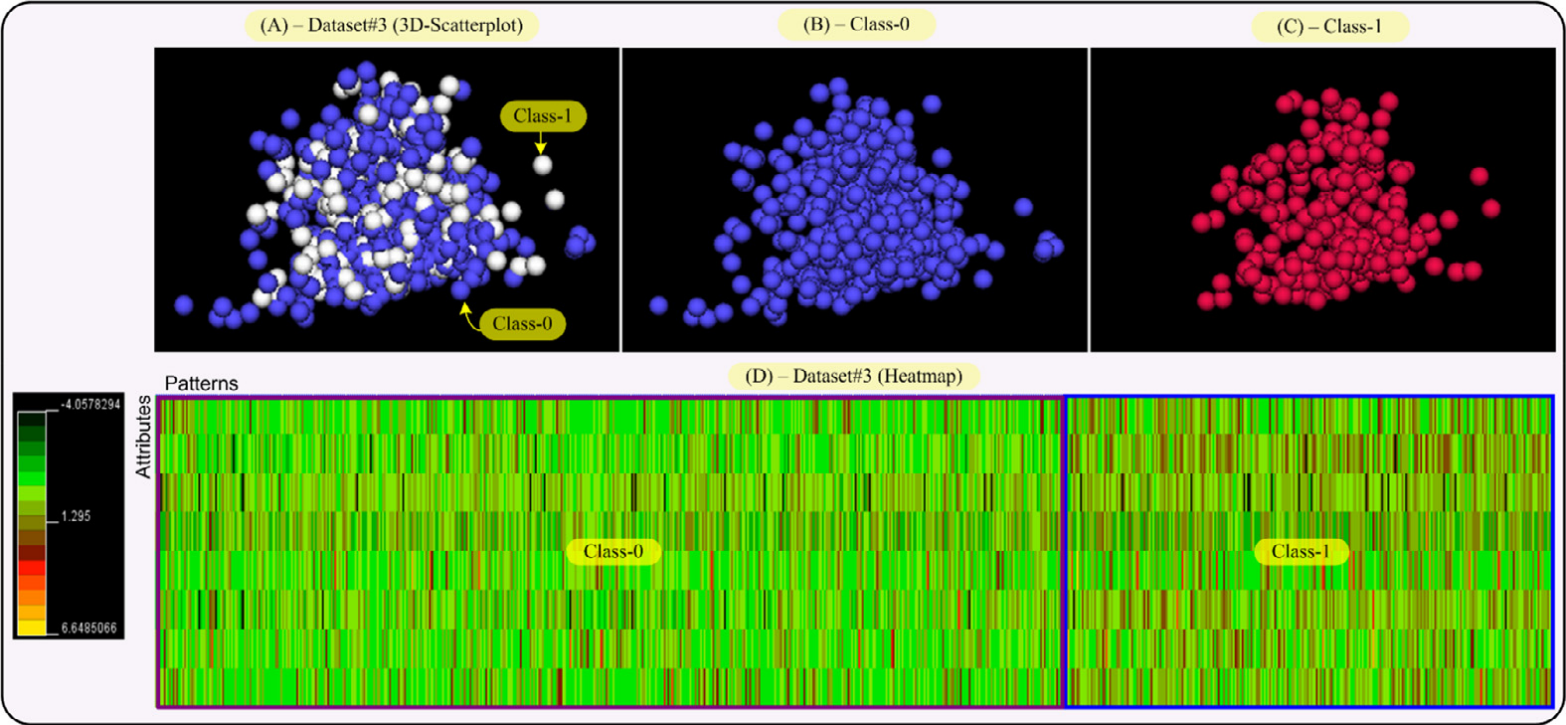
3D-scatterplot and heatmap visualizations for Dataset#3 (Diabetes Data). Each class in the scatterplot has a different color and the color scale for the heatmap goes from dark green (value: −4.06), passing by red (value: 1.29), to yellow (value: 6.65). Each class in Dataset#3 has also been framed on the heatmap. (For interpretation of the references to color in this figure legend, the reader is referred to the web version of this article.)

Fig. 5 shows the 3D-scatterplot and heatmap for Dataset#3. As displayed in the scatterplot, Dataset#3 is a compact dataset and data in each class are inter-related as those in Fig. 4. According to the heatmap of Dataset#3, we have that both classes are much more interlinked than the classes in Dataset#2, which becomes the task of separating such classes much more complex. Hence, RIM-GP classifiers from Dataset#3 have reached the greatest number of rules per classifier and patterns at the intersection (see Table B.2 in the paper) in comparison with the remaining data sets. Then, we have that RIM-GP classifiers have reached the worst accuracy results from Dataset#3 (with a mean accuracy value of 61.35%).

**Fig. 6:**
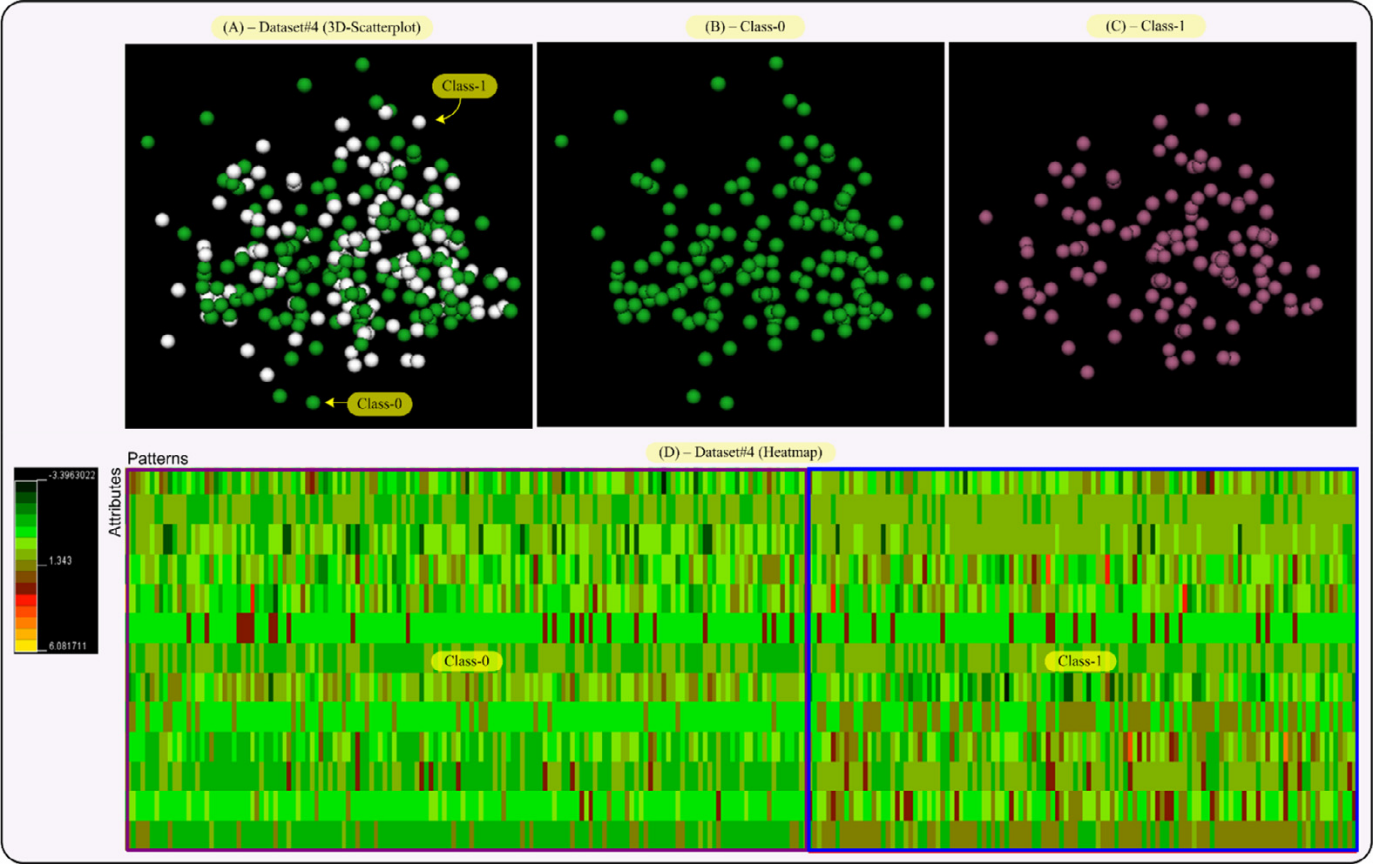
Fig. 6. 3D-scatterplot and heatmap visualizations for Dataset#4 (Statlog Heart Data). Each class in the scatterplot has a different color and the color scale for the heatmap goes from dark green (value: −3.39), passing by red (value: 1.33), to yellow (value: 6.07). Each class in Dataset#4 has also been framed on the heatmap. (For interpretation of the references to color in this figure legend, the reader is referred to the web version of this article.)

Fig. 6 shows the 3D-scatterplot and heatmap for Dataset#4. Note that the profile of views in this figure is similar to that given in Fig. 4. Such a similarity could be associated with the fact that both data sets (Dataset#2 and Dataset#4) come from the same disease domain, i.e., heart disease data, although they come from different data sources, which make them different. One of the visual differences is that pattern-points in the scatterplot of Fig. 6 are more scattered than those of Fig. 4. Then, since the profile of views in both figures is similar, it is expected that the RIMGP results on Dataset#2 be similar to the ones on Dataset#4, which is true because the results for Dataset#2 and Dataset#4 given in Table B.2 of the paper have similar values.

Concluding on interpretation of results based on data visualization, we can say that the given visualizations correspond to the results reached by RIM-GP. In other words, they explain the results and have played a major role in their understanding. Therefore, the use of this kind of visualizations can be very useful to predict in advance, possible behaviors of methods (as RIM-GP) from data sets.

## Declaration of Competing Interest

The authors declare that they have no known competing financial interests or personal relationships that could have appeared to influence the work reported in this paper.
